# Response to the challenges of pandemic H1N1 in a small island state: the Barbadian experience

**DOI:** 10.1186/1471-2458-10-S1-S10

**Published:** 2010-12-03

**Authors:** Natasha Sobers-Grannum, Karen Springer, Elizabeth Ferdinand, Joy St John

**Affiliations:** 1 Ministry of Health, Frank Walcott Building, Culloden Road, St. Michael, Barbados

## Abstract

**Background:**

Having been overwhelmed by the complexity of the response needed for the severe acute respiratory syndrome (SARS) epidemic, public health professionals in the small island state of Barbados put various measures in place to improve its response in the event of a pandemic

**Methods:**

Data for this study was collected using Barbados’ National Influenza Surveillance System, which was revitalized in 2007. It is comprised of ten sentinel sites which send weekly notifications of acute respiratory illness (ARI) and severe acute respiratory illness (SARI) to the Office of the National Epidemiologist. During the 2009 H1N1 pandemic, meetings of the National Pandemic Planning Committee and the Technical Command Committee were convened. The pharmaceutical and non-pharmaceutical interventions (NPIs) implemented as a result of these meetings form the basis of the results presented in this paper.

**Results:**

On June 3, 2009, Barbados reported its first case of 2009 H1N1. From June until October 2009, there were 155 laboratory confirmed cases of 2009 H1N1, with one additional case occurring in January 2010. For the outbreak period (June-October 2009), the surveillance team received reports of 2,483 ARI cases, compared to 412 cases for the same period in 2008. The total hospitalization rate due to SARIs for the year 2009 was 90.1 per 100,000 people, as compared to 7.3 per 100,000 people for 2008. Barbados’ pandemic response was characterized by a strong surveillance system combining active and passive surveillance, good risk communication strategy, a strengthened public and private sector partnership, and effective regional and international collaborations. Community restriction strategies such as school and workplace closures and cancellation of group events were not utilized as public health measures to delay the spread of the virus. Some health care facilities struggled with providing adequate isolation facilities.

**Conclusions:**

The number of confirmed cases was small but the significant surge in ARI and SARI cases indicate that the impact of the virus on the island was moderate. As a result of 2009 H1N1, virological surveillance has improved significantly and local, regional and international partnerships have been strengthened.

## Background

Pre-pandemic influenza preparedness is regarded as a critical function of public health [1] but in the midst of difficult economic conditions it can present a significant challenge to both developed and developing nations. To effectively detect a potential pandemic in the earliest possible stage, international health organizations recognized the need for all countries, regardless of size, to develop sensitive surveillance systems to be able to detect the entry of novel viruses into the population [2].

Barbados is the most easterly island in the Caribbean Sea, measuring 166 square miles with an estimated mid-year population in 2009 of 275,719 [3]. Barbados’ per capita GDP was USD 11.9 thousand in 2009, and approximately eleven percent of total government expenditure is spent on health care [4]. Primary health care is available to all citizens free of charge through government run community based health centres (polyclinics). Alternatively, persons may also access primary health care through a thriving private sector. In 2009, the island was regarded as having a very high human development, ranking 37^th^ on the Human Development Index scale [5].

Having been overwhelmed by the complexity of the response needed for the severe acute respiratory syndrome (SARS) epidemic, Barbadian public health professionals put various measures in place to improve its response in the event of a pandemic. In accordance with the resolution at the 58^th^ World Health Assembly (WHA) entitled *Strengthening Pandemic Influenza Preparedness and Response*[6], Barbados developed a National Influenza Pandemic Preparedness Plan (NIPPS) in August 2006 [7]. In September 2007, a pandemic flu outbreak training workshop was held and a pandemic manual was subsequently developed. This manual was later revised by a team of managers of the public community health centeres, and a two day seminar was held in April 2009 for private and public sector health care professionals to launch this protocol and to educate participants regarding the appropriate response to dangerous infectious diseases. These measures were accomplished through technical cooperation with the Pan American Health Organization (PAHO) and the Caribbean Epidemiological Centre (CAREC).

**Figure 1:**
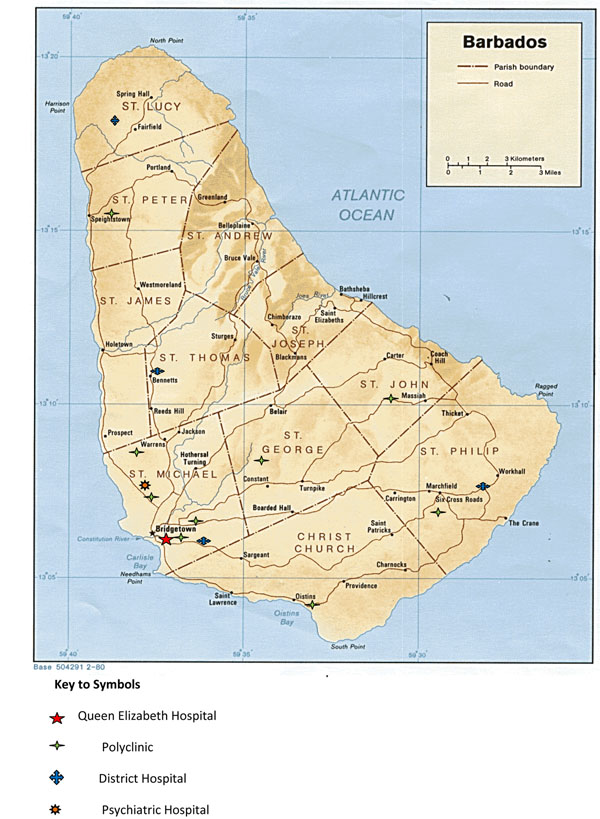
Map of Barbados with location of public hospital and community-based health centres (polyclinics).

In 2007, the National Influenza Surveillance System was revitalized beginning with surveillance of cases of acute respiratory illness (ARI) at the countries eight community health centres which served as sentinel sites. These sentinel sites (polyclinics) are located at strategic points across the island (Figure 1). This was expanded in January 2008, to include the island’s lone tertiary public hospital where cases of severe acute respiratory illness (SARI) are detected routinely through active surveillance.

Barbados’ NIPPS plan follows international guidelines with recommendations for both pharmaceutical and non-pharmaceutical interventions to be implemented at various stages of a pandemic. In April 2009, when the World Health Organization (WHO) announced that the world was experiencing an influenza pandemic, Barbadian public health officials responded to the threat. In this paper, we examine the response of public health professionals in implementing plans regarded as best practice for developed nations and consider the peculiarities of implementation in a small island state.

## Methods

Data for this study was collected using Barbados’ National Influenza Surveillance System which is comprised of ten sentinel sites, responsible for sending weekly notifications to the Ministry of Health of ARI and SARI Using guidelines provided CAREC [8], a case was reported as an ARI if it met the following case definition: acute (sudden) febrile illness (>38.0°C or 100.4°F) in a previously healthy person, presenting with cough or sore throat with or without respiratory distress. Cases were reported as SARI if they presented a sudden onset of fever over 38°C, cough or sore throat, shortness of breath or difficulty breathing, and required hospital admission.

During the pre-pandemic period, as part of routine surveillance, nasopharyngeal swabs were taken from all cases of SARI detected at the hospital sentinel site and a sample of six swabs from patients meeting the criteria of ARI from two of the most centrally located ambulatory sites. In April 2009, after the announcement by the WHO that the world had entered pandemic phase five, an enhanced testing strategy was introduced and all primary health care facilities, both private and public, were asked to take nasopharyngeal swabs from all persons who presented with fever (>38°C) with respiratory symptoms and a travel history to an affected area. When sustained community transmission of 2009 H1N1 was established, this testing strategy was returned to the pre-pandemic level.

Nasopharyngeal samples taken from suspected cases were sent first to the Barbados Public Health Laboratory (local) where they underwent preliminary screening using immunofluorescence testing. Using this method, it is possible to detect influenza A virus, adenovirus, respiratory syncitial virus, parainfluenza types 1, 2 and 3 and influenza B. All samples which met the criteria for testing, irrespective of result, were sent to CAREC. At the peak of the epidemic in the Caribbean, Barbadian health officials began sending some samples to the U.S. Centers for Disease Control and Prevention (CDC) in Atlanta, Georgia in an attempt to reduce the burden being placed on CAREC. The CDC and CAREC collaborated during the outbreak to provide critical guidance and technical capacity to the region.

During the pandemic, the Ministry of Health’s public health officials convened meetings of the National Pandemic Planning Committee which met at least weekly for the first two months of the declaration of a pandemic and then monthly for the duration of the outbreak in Barbados. A smaller Technical Command Committee was also convened to manage the response to the pandemic and met weekly. At the end of the outbreak period in Barbados, a formal evaluation was conducted by many of the major stakeholders within the health sector. The pharmaceutical and non-pharmaceutical interventions (NPIs) implemented as a result of these meetings form the basis of the results presented in this paper.

The evidence surrounding the use of some NPIs to delay spread of infection in a pandemic has been found to be weak [9],[10],[11]. Aledort et al. published a systematic review which examined the literature and also made recommendations based on expert opinion in cases where there were no or very low quality articles available as a study. Here we consider the pharmaceutical and non-pharmaceutical interventions carried out by the Government of Barbados through the Ministry of Health, and compare these interventions to the recommendations of the article by Aledort et al [9].

## Results

### Overview of cases

**Figure 2:**
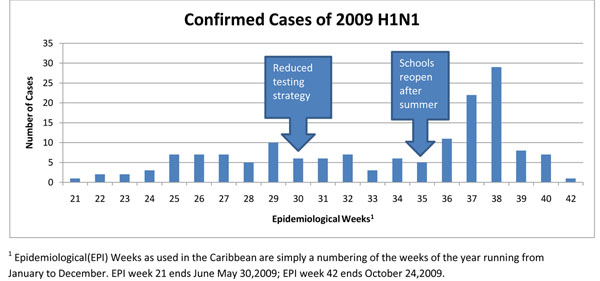
**Laboratory confirmed cases of 2009 H1N1 by epidemiological week**. ^1^Epidemiological (EPI) Weeks as used in the Caribbean are simply a numbering of the weeks of the year running from January to December. EPI week 21 ends May 30, 2009; EPI week 42 ends October 24, 2009.

On June 3, 2009, Barbados reported its first case of 2009 H1N1. From June until October 2009 there were 155 confirmed cases of 2009 H1N1 (Figure 2). Since October 24, 2009, there has only been one confirmed case of 2009 H1N1, which occurred in January 2010. The cases range in age from 23-days-old to 65-years-old, with a mean age of 17-years-old; the greatest proportion of our cases occurred in the 5-14 age group and the second highest in the 15-24 age group. A little more than half (53.5%) of all confirmed 2009 H1N1 viral infections occurred in females. The most common presenting symptoms were fever - 92.9% (144 cases); and cough or sore throat - 82.6% (128 cases). Only 35.5% (55) of cases presented with gastrointestinal symptoms. Of the 155 confirmed cases, there were three fatalities, which occurred in persons with underlying chronic conditions, all of whom were morbidly obese.

For the outbreak period (June to October 2009), the surveillance team received reports of 2,483 cases, compared to 412 cases for the same period in 2008. There were 179 SARI cases from June to October 2009, 6% (10) of which required ventilation and care in the intensive care unit. During this time there were seven SARI deaths. Of these, four received nasopharyngeal swabs that were tested for 2009 H1N1 and three tested positive.

The total hospitalization rate due to SARIs for the year 2009 was 90.1 per 100,000 people, compared to 7.3 per 100,000 people for 2008. The highest hospitalization rate occurred in children less than one year (400 per 100,000) followed by those 1 to 4 years old (290 per 100,000).

### Non-pharmaceutical interventions

#### Human surveillance

##### Case reporting and early rapid viral diagnosis

During the initial phases of the pandemic while knowledge of the virus’ characteristics was limited, all suspected cases in the island were reported to the Office of the National Epidemiologist and nasopharyngeal swabs taken. All cases suspected of having 2009 H1N1 were investigated and close contacts monitored until the results of the swab were obtained. As the outbreak advanced, only laboratory-confirmed cases and suspected hospitalized cases were reported. Immunoflourescent testing was done on the swabs in country to test for influenza A virus, but this test was incapable of subtyping and thus swabs had to be sent to a regional centre for real-time polymerase chain reaction testing to be done. This resulted in wait times for results that averaged one week but were occasionally as long as six weeks. Rapid testing was not utilized in Barbados.

##### Hand hygiene, respiratory etiquette and disinfection

The Ministry of Health placed great emphasis on hand hygiene and respiratory etiquette in its communication messages to the public. The *WHO Outbreak Communication Guidelines*[12] were used as the risk communication guide in responding to the emergence of 2009 H1N1 in our community. These guidelines use trust, early announcements, transparency, listening and planning as key components of risk communication [12]. Several protocols were distributed on hand hygiene to schools, day care centres, workplaces and the general public. An infectious waste protocol was developed to guide health facilities in the disposal of infectious waste.

##### Surgical and N95 masks and other Personal Protective Equipment

Personal Protective Equipment (PPE) was donated by the U.S. Agency for International Development (USAID) in May 2008 and USD 40,500 in supplies was approved for the financial year (2008/2009) and utilized in 2009/2010. During the pandemic large amounts of PPE were used in both the public and private sector and a protocol governing distribution and usage was developed and circulated. The central storage facility has been improved upon during this time but remains challenged by lack of security to prevent theft and insufficient human resources for efficient stock-taking.

#### Patient management

##### Isolation of sick individuals

As part of their efforts towards pandemic preparedness, the Ministry of Health in Barbados held a seminar in April 2009, at which they disseminated a manual on management of Dangerous Infectious Diseases to middle- and senior-level managers of at least 90% of health care facilities in the country. This manual provided detailed instructions to health care leaders on the structure and type of isolation facilities that ought to be available at their facility.

During the outbreak, health care facilities attempted to follow these evidence-based guidelines but were challenged in some regards by their existing structures and layout, and restricted by the high costs that would have been necessary to change these facilities. The island’s lone public hospital is the only major health centre with designated isolation facilities but its capacity was significantly overwhelmed during the outbreak. The community health centres created temporary isolation areas by reorganizing, and in some cases, curtailing routine services. Administrators and health care providers remained committed to the principles of patient isolation for dangerous infectious diseases and have stated their intention to revise their protocols so that there are evidence-based and yet feasible and practical for each facility.

#### Contact management

##### Quarantine and contact tracing

Ministry of Health officials took the decision early in the pandemic that there was insufficient evidence to support quarantining of asymptomatic persons who had been in contact with a probable or confirmed case or had travelled to an affected area internationally. The protocol adopted for contact tracing varied according to whether persons were regarded as probable or confirmed cases.

A *probable case* is an individual with an influenza test that is positive for influenza A, but is unsubtypable by reagents used to detect seasonal influenza virus infection, or an individual with a clinically compatible illness or who died of an unexplained acute respiratory illness, and who is considered to be epidemiologically linked to a probable or confirmed case.

A *close contact* is an individual who has cared for, lived with or had direct contact with respiratory secretions or body fluids of a probable or confirmed case of influenza A/H1N1. For probable cases, close contacts were followed at home and work. Contact tracing was coordinated by the Medical Officer of Health (community-based public health leader) and a team operating within the community. Close contacts with symptoms were isolated at home or in hospital depending on the severity of symptoms. Contacts were given a short sensitization session and fact sheets on hand hygiene, respiratory etiquette and proper cleaning methods of laundry and other household items.

#### Community restrictions

##### School and workplace closures

At the peak of the epidemic in Barbados, many primary (ages 5-11) and secondary schools (ages 11-18) reported absenteeism rates from schools ranged from as low as 9% to as high as 40%. Based on the latest available evidence, the Ministry of Health, in collaboration with Ministry of Education, decided not to close schools in hope of preventing further spread because the benefit of doing so was not sufficient enough to justify the social and economic consequences of such an action. There was still, however, some disruption within schools. At the start of the pandemic each school that was affected through infection by either students or teachers, was visited by public health officials to educate and allay fears of mass morbidity and mortality. This meant that classes were cancelled for approximately 1-2 hours in each case as fears were addressed. Public health officials also visited the workplaces of the first reported cases to conduct similar educational seminars, so some productivity would have been lost during that time. One school, however, reported high (75%) absenteeism among staff , which resulted in education officials making the decision to close the school to prevent issues of discipline and security from arising.

##### Cancellation of group events

The ‘Crop Over Festival’ is Barbados’ major cultural extra vaganza for the calendar year and is a significant source of revenue for the island. The festival is held from July to August each year and is characterized by social gatherings throughout the season, which may range from 100 to 30,000 persons. Given the available evidence, the decision was taken not to cancel any of the events associated with the festival, but ill persons were asked not to attend the gatherings. Patrons were asked to refrain from their usual custom of waving rags and using shared drink containers. The festival activities were used to educate the populace in the use of appropriate hand hygiene and respiratory etiquette. This education was done using calypso jingles that represent the signature musical genre of the festival, as well as through distribution of flyers along the highways as persons engaged in the festivities.

### Pharmaceutical intervention methods

#### Pharmaceuticals – oseltamivir

The Barbados Drug Service was able to procure 49,000 courses of oseltamivir (Tamiflu) as part of pre-pandemic preparedness. A protocol was developed by the Ministry of Health to manage the distribution of Tamiflu in both the private and public sector. This protocol was first circulated in May 2009, and use was restricted to those with moderate to severe respiratory illness who met the case definition of a suspected case, which at that time included fever, cough and/or sore throat and a travel history to an affected area. As the disease became more widespread in Barbados, the case definition for a suspected case of H1N1 was modified to exclude the travel requirement, and Tamiflu usage was thus increased. As more information became available about the virus, the protocol was revised; in July 2009 those with mild respiratory illness who had certain specified chronic diseases and those with moderate to severe illness were eligible to receive Tamiflu. The drug was widely used throughout the outbreak and no cases of resistance were reported.

#### Pharmaceuticals – vaccine

Plans for procurement of 2009 H1N1 vaccine were made through the Revolving Fund of the Pan American Health Organization. A conference of the *Sub-Regional Workshop for the Planning of Pandemic Vaccine Introduction* was attended by Ministry of Health officials to develop a plan for the deployment of the vaccine within two to four weeks after its arrival on the island. The plan, which was based on a PAHO vaccination guide [13], identified health care workers, pregnant women, and persons over six months with underlying diseases as the main target groups for vaccination. The initial target was 50,000 doses based on estimations of prevalence of the diseases in the Barbadian population. Due to economic constraints and estimates of anticipated vaccine uptake, the actual number of doses acquired by the government was 20,000 doses at a cost of approximately USD 150,000. This cost includes only that of the actual vaccine and excludes the extra supplies and human resources that would be needed to administer the vaccine. The vaccination campaign began in February 2010. After four weeks, 39% of the estimated target group had been reached—51% of health care workers, 10% of pregnant women and 31% of persons who had been targeted with chronic disease. The vaccine campaign was extended for a further 6 months; 10,900 (54%) doses of the vaccine have been utilized.

## Discussion

Generally, public health leaders in Barbados responded quickly and decisively to the threat of pandemic 2009 H1N1. Protocols were developed, disseminated and adhered to in the majority of the private and public sector. The response was characterized by technical cooperation between public and private sector within the country as well as regional (PAHO and CAREC) and extra-regional (CDC) alliances. The risk communication techniques employed served to construct and reaffirm partnerships and reassure the Barbadian public. One local newspaper produced a headline at the start of the outbreak remarking on the public’s “Calm Response to H1N1” [14].

Most of the non-pharmaceutical interventions employed (Table 1) closely followed recommendations made by international organizations such as the WHO and CDC [15],[16],[17] For example, hand hygiene and respiratory etiquette which received the strongest evidence in the scientific literature [18],[19],[20],[21] formed the foundation of Barbados’ pandemic response.

**Table 1:** Non-pharmaceutical intervention methods used in Barbados’ response to H1N1

		Used in Barbados	

Non-pharmaceutical Intervention	Recommended for Use	Early localized	Advanced
Human Surveillance			
Case reporting	Yes	Yes	Yes
Early rapid viral diagnosis	Yes	No	No
Disinfection	No	No	No
Hand Hygiene	Yes	Yes	Yes
Respiratory etiquette	Yes	Yes	Yes
Surgical and N95 Masks for general public	Inconclusive	Yes	Yes
Other personal protective equipment	No	Yes	No
Screening of travellers from affected international region	Inconclusive	Yes	No
Patient Management			
Isolation of sick individuals	Yes	Yes	Yes
Provision of social support services to the isolated	Yes	Yes	No
Contact Management			
Quarantine	Inconclusive	No	No
Contact tracing	Inconclusive	Yes	No
Community Restrictions			
School Closures	No	No	Yes
Workplace closures	No	No	No
Cancellation of group events	No	No	No
International and domestic travel restrictions	No	No	No

For interventions with less conclusive scientific evidence, social and economic factors weighed heavily in deciding whether or not to include them. The use of rapid tests in the pre-pandemic and early pandemic phases was recommended Aledort et al [9]. However, the recommendation was made with the reservation that these tests often have suboptimal sensitivity [9,22]. Several other sources advised against the use of these tests [16]. In Barbados, having weighed the benefits of rapid diagnosis against the high costs and wide margins of error, the use of rapid tests was decided against.

Aledort et al. recommended against the use of surgical and N95 masks for the general public at all pandemic phases with the exception of the advanced stage where it is stated that the evidence was inconclusive [9]. However, Jeff erson et al. have shown that in health care settings, the use of masks could reduce the transmission of influenza [23]. In Barbados’ response, persons entering health care facilities such as the polyclinics were asked to wear surgical masks.

It is difficult to determine the true impact of 2009 H1N1 as compared to regular seasonal influenza in the island since the National Surveillance System is still relatively new. In fact, virological surveillance was practically non-existent prior to the announcement of pandemic phase five. This component of surveillance was present in the protocol but lacked sufficient physician motivation and thus Ministry of Health officials used the opportunity of the emerging virus to encourage the taking of nasopharyngeal swabs.

## Conclusions

The number of confirmed cases was small, but the significant surge in ARI and SARI cases noted at the sentinel sites indicate that the impact of the virus on the island was moderate. Barbados enjoyed excellent political commitment to the executing of its pandemic plan but was challenged by limited financial resources. As a result of 2009 H1N1, virological surveillance has improved significantly and local, regional and international partnerships have been forged and in some cases strengthened.

## Abbreviations

ARI: Acute respiratory infections; CAREC: Caribbean Epidemiological Centre; CDC: U.S. Centers for Disease Control and Prevention; GDP: Gross Domestic Product; ICU: Intensive Care Unit; NIPPS: National Infuenza Pandemic Preparedness Plan; NPI: Non-pharmaceutical interventions; PAHO: Pan American Health Organization; PPE: Personal Protective Equipment; SARI: Severe acute respiratory infection; SARS: Severe acute respiratory syndrome; USAID: United States Agency for International Development; WHA: World Health Assembly; WHO: World Health Organization.

## Competing interests

The authors would like to state that they have no competing interests.

## Authors’ contributions

NSG made substantial contributions to the acquisition of, analysis and interpretation of data and was responsible for drafting the manuscript. EF, KS and JSJ contributed to the conception and design of the study. All authors were involved in revising it critically for important intellectual content and have approved the final version of this publication.

## References

[B1] Pan American Health Organization López-Acuña D Public Health in the Americas 2002 Washington DC

[B2] World Health Organization Strengthening pandemic-influenza preparedness and response, including application of the International Health Regulations (2005) Fifty-Ninth World Health Assembly 2006 Geneva, Switzerland

[B3] Barbados Statistical Service http://www.barstats.gov.bb

[B4] Government of Barbados Ministry of Economic Affairs Barbados Economic and Social Report 2009 2010 Bridgetown

[B5] Statistics/ Human Development/United Nations Development Program http://hdr.undp.org/en/statistics

[B6] World Health Organization Strengthening Pandemic Influenza Preparedness and Response Fifty-eighth World Health Assembly 2005 Geneva, Switzerland

[B7] Harding SB Carter-Taylor D Chapman R Deane S Barbados National Influenza Pandemic Preparedness Plan 2006 (Unpublishedreport)

[B8] Caribbean Epidemiological Centre Regional Communicable Disease Surveillance Systems for CAREC Member Countries - Policy Guidelines 2008 Port of Spain

[B9] Aledort JE Lurie N Wasserman J Bozzette SA Non-pharmaceutical public health interventions for pandemic influenza: an evaluation of the evidence base BMC Public Health 2007 7 208 17697389 10.1186/1471-2458-7-208 PMC2040158

[B10] Bell DM Non-pharmaceutical interventions for pandemic influenza, national and community measures Emerg Infect Dis 2006 12 1 88 94 16494723 10.3201/eid1201.051371 PMC3291415

[B11] Vukotich CJ Jr Coulborn RM Aragon TJ Baker MG Burrus BB Aiello AE Findings, gaps, and future direction for research in nonpharmaceutical interventions for pandemic influenza [conference summary] Emerg Infect Dis [serial on the Internet] 2010 Retrieved from internet September 2010 10.3201/eid1604.090719 20350370

[B12] World Health Organization WHO Outbreak Communication Guidelines 2005 Geneva

[B13] Pan American Health Organization TAG final recommendations on pandemic influenza 2009 Costa Rica

[B14] Moore T Calm Response to H1N1 Nation Newspaper 2009 Bridgetown: Nation Publishing Company

[B15] H1N1 Flu: Infection Control http://www.cdc.gov/h1n1flu/infectioncontrol

[B16] Interim Guidance for the Detection of Novel Influenza A Virus Using Rapid Influenza Diagnostic Tests http://www.cdc.gov/h1n1flu/guidance/rapid_ testing.htm

[B17] World Health Organization WHO recommendations on the use of rapid testing for influenza diagnosis http://www.who.int/csr/disease/avian_influenza/guidelines/RapidTestInfluenza_web.pdf

[B18] Boyce JM Pittet D Guideline for Hand Hygiene in Health-Care Settings. Recommendations of the Healthcare Infection Control Practices Advisory Committee and the HIPAC/SHEA/APIC/IDSA Hand Hygiene Task Force Am J Infect Control 2002 30 8 S1 46 10.1067/mic.2002.130391 12461507

[B19] Aiello AE Murray GF Perez V Coulborn RM Davis BM Uddin M Shay DK Waterman SH Monto AS Mask use, hand hygiene, and seasonal influenza-like illness among young adults: a randomized intervention trial J Infect Dis 201 4 491 498 10.1086/650396 20088690

[B20] U.S. Centers for Disease Control and Prevention Respiratory hygiene/cough etiquette in healthcare settings http://www.cdc.gov/flu/professionals/infectioncontrol/resphygiene.htm

[B21] Aiello AE Coulborn RM Perez V Larson EL Effect of hand hygiene on infectious disease risk in the community setting: a meta-analysis Am J Public Health 2008 98 8 1372 1381 18556606 10.2105/AJPH.2007.124610 PMC2446461

[B22] Cazacu AC Chung SE Greer J Demmler GJ Comparison of the directigen flu A+B membrane enzyme immunoassay with viral culture for rapid detection of influenza A and B viruses in respiratory specimens J Clin Microbiol 2004 42 8 3707 3710 15297520 10.1128/JCM.42.8.3707-3710.2004 PMC497654

[B23] Jefferson T Foxlee R Del Mar C Dooley L Ferroni E Hewak B Prabhala A Nair S Rivetti A Physical interventions to interrupt or reduce the spread of respiratory viruses: systematic review BMJ 2008 336 7635 77 80 18042961 10.1136/bmj.39393.510347.BE PMC2190272

